# Red Complex Pathogens, Periodontal Dysbiosis and Periodontal Therapy in Alzheimer’s Disease and Dementia

**DOI:** 10.3390/jcm15135296

**Published:** 2026-07-07

**Authors:** Julia Bijoch, Karol Jędrasiak

**Affiliations:** 1 Collegium Medicum, Faculty of Medicine, WSB University, 41-300 Dabrowa Gornicza, Poland; 2 Department of Transport and Computer Science, WSB University, 41-300 Dabrowa Gornicza, Poland; kjedrasiak@wsb.edu.pl

**Keywords:** periodontal dysbiosis, red complex pathogens, *Porphyromonas gingivalis*, periodontitis, Alzheimer’s disease, dementia

## Abstract

This narrative review synthesizes evidence linking periodontal dysbiosis with Alzheimer’s disease, all-cause dementia, and dementia-relevant mechanisms, focusing on the red complex pathogens *P. gingivalis*, *T. denticola*, and *T. forsythia* and on the translational meaning of periodontal therapy. A PubMed-centered literature search up to May 2026 informed this synthesis (a narrative review, not a registered systematic review or meta-analysis) of 46 periodontal-scope sources, supplemented by five contextual references identified outside the periodontal search (51 references in total). *P. gingivalis* shows the strongest mechanistic support, including gingipains, lipopolysaccharide, outer membrane vesicles, endothelial stress, and neuroinflammatory signaling; *T. denticola* shows moderate biological plausibility; and *T. forsythia* remains mainly hypothesis-generating. Human studies associate periodontitis, tooth loss, oral-hygiene indicators, and periodontal-care exposure with dementia-relevant outcomes, but residual confounding, reverse causation, dental-care access, and heterogeneous endpoints preclude causal inference. Notably, direct targeting of a single periodontal pathogen has not shown clinical benefit, as the gingipain inhibitor atuzaginstat failed in the GAIN trial, contrasting with the modest success of amyloid-targeting therapies. Current evidence supports graded plausibility rather than causal certainty, and a registered multi-database systematic review with neurological endpoints is needed before meta-analytic clinical claims can be made.

## 1. Introduction

Periodontitis is a chronic biofilm-driven inflammatory disease in which dysbiotic subgingival communities interact with host susceptibility and progressively destroy tooth-supporting tissues. Its potential relevance for brain aging is biologically plausible because ulcerated periodontal pockets may release bacteria, bacterial products, inflammatory mediators, and extracellular vesicles into the circulation. The public health context is substantial, since the World Health Organization estimates that oral diseases affect almost half of the global population and that severe periodontal disease affects about 1 billion people [1]. Dementia is similarly a major global health challenge, with rising prevalence projected across world regions and substantial disparities in diagnosis, care, and research capacity [2]. Contemporary prevention frameworks emphasize that late-life cognitive decline results from multiple interacting biological, vascular, social, and behavioral risk processes. It should be noted that periodontitis and oral health are not currently listed among the modifiable dementia risk factors in the 2024 Lancet Commission report; the present review therefore treats oral health as an emerging, biologically plausible candidate rather than an established modifiable risk factor [3].

The red complex concept remains a useful historical and ecological entry point, although it should not be treated as a complete modern microbiome model. Socransky and colleagues grouped *P. gingivalis*, *T. denticola*, and *T. forsythia* as organisms associated with advanced periodontal destruction [4]. Contemporary oral microbiome research shows that periodontitis is better understood as a dysbiotic consortium and host-response disorder rather than a single-pathogen infection. This distinction is essential for neurodegeneration research because a biologically plausible oral–brain axis may operate through microbial communities, virulence factors, host inflammation, vascular pathways, and behavioral confounding rather than through one organism alone.

This review therefore uses a deliberately graded interpretation. *P. gingivalis* is treated as the best-developed mechanistic candidate, *T. denticola* as a biologically credible but incompletely validated candidate, and *T. forsythia* as a periodontal inflammatory organism with mainly indirect neurobiological relevance. The clinical question is also separated from the mechanistic question. Even if periodontal pathogens can affect barrier cells, immune activation, or neural tissues in experimental systems, this does not prove that periodontal treatment prevents Alzheimer’s disease or dementia in humans.

## 2. Materials and Methods

This narrative review examines two related questions: first, whether periodontitis and the underlying periodontal dysbiosis, including exposure to red complex pathogens, are associated with Alzheimer’s disease, all-cause dementia, cognitive decline, or dementia-relevant biological markers, and second, whether periodontal treatment modifies these outcomes. Parkinson’s disease and other neurodegenerative disorders are discussed only as contextual evidence when they inform barrier biology, oral microbial translocation, or broader neuroinflammatory plausibility.

This work is a narrative review. It was not prospectively registered, did not use duplicate independent screening, did not search multiple bibliographic databases, and did not include a formal meta-analysis. Sources were selected for their relevance to the thematic argument rather than through formal eligibility screening. The manuscript should therefore not be interpreted as a registered or Preferred Reporting Items for Systematic Reviews and Meta-Analyses (PRISMA)-compliant systematic review.

PubMed was searched up to 4 May 2026 using twelve query groups covering oral microbiome, oral microbiota, periodontitis, periodontal disease, *P. gingivalis*, gingipains, *T. denticola*, *T. forsythia*, red complex organisms, periodontal pathogens, dementia, Alzheimer’s disease, Parkinson’s disease, cognitive decline, neuroinflammation, periodontal therapy, periodontal instrumentation, host modulation, and local pharmacological adjuncts. Records returned by these query groups were reviewed by the authors for relevance to the oral–periodontal–neurodegeneration scope, and sources were then selected purposively to represent the mechanistic, observational, and intervention-adjacent evidence most relevant to the review’s two guiding questions.

**Figure 1:**
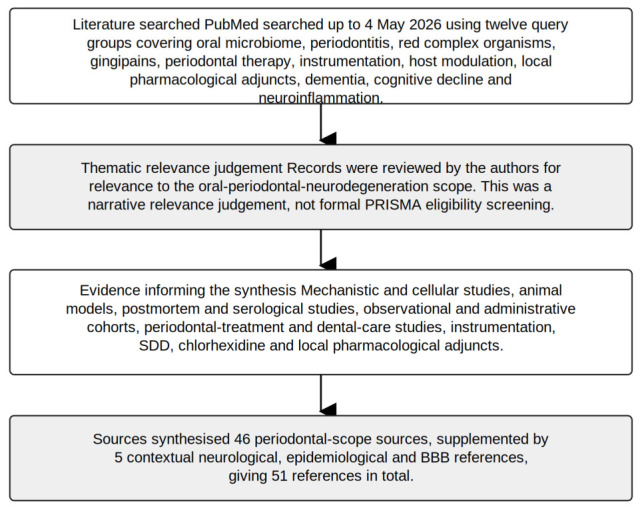
Overview of the literature search and narrative source selection. The PubMed query groups were screened by the authors for thematic relevance, and sources were selected purposively to inform the synthesis. This is not a PRISMA flow diagram and does not represent formal eligibility screening, duplicate independent screening, or multiple-database coverage. Full query groups and the narrative source-selection log are provided within the manuscript in Appendix A and Table A1.

Sources were judged to be relevant when they addressed periodontitis, oral microbial dysbiosis, red complex organisms, periodontal treatment, dental-care exposure, or periodontal inflammatory burden in relation to Alzheimer’s disease, dementia, cognitive decline, neuroinflammation, blood–brain barrier biology, or brain-relevant microbial mechanisms. Topics outside this scope, non-periodontal oral conditions without mechanistic relevance, acute infection alone, unrelated neurological disease, and purely technical microbiome work without brain-relevant interpretation, were not discussed. Forty-six periodontal-scope sources inform the synthesis; most are cited inline in the main narrative, while the remainder are methodological, tabular, and background references that support the interpretive framework and red complex virulence context. Five additional contextual references identified outside the periodontal literature—two pivotal Alzheimer’s disease therapeutic trials, one dementia-epidemiology reference, and two references on matrix-metalloproteinase and blood–brain barrier biology—are cited for essential clinical, epidemiological, and mechanistic context, giving 51 references in total. The scope of the review and the key interpretive distinctions are summarized in Table 1. The complete PubMed query groups, search date, source-selection counts and narrative screening log are provided within the manuscript in Appendix A and Table A1. An overview of the literature search and narrative source-selection process is shown in Figure 1. Because this review is a narrative synthesis, that log is a transparency instrument rather than a PRISMA eligibility flow or a duplicate-screened extraction file.

For clinical and intervention-adjacent evidence, we considered potential sources of bias narratively—confounding, participant selection, exposure or intervention classification, missing data, outcome measurement, and selective reporting—to discipline interpretation. Studies were read with greater caution when treatment exposure could plausibly reflect dental-care access, education, socioeconomic status, baseline cognition, comorbidity burden, medication use, health literacy, caregiver support, or adherence behavior.

Evidence was not pooled because exposure definitions, microbial assays, dementia ascertainment, periodontal treatment categories, imaging endpoints, inflammatory endpoints, and follow-up duration were heterogeneous. In particular, the periodontal-treatment signal reported by Schwahn et al. [5] used an Alzheimer’s disease-related brain-atrophy estimate, whereas Qi et al. [6] reported dementia incidence using a hazard ratio. These outcomes are not statistically commensurable without a predefined transformation framework, harmonized target estimand, and study-level variance extraction. Clinical conclusions are therefore expressed as graded plausibility statements rather than pooled effect estimates.

## 3. Periodontal Red Complex Biology and Its Relevance to Brain Aging

*P. gingivalis* has the strongest mechanistic support within the periodontal brain hypothesis. Its gingipains, lipopolysaccharide (LPS), and outer membrane vesicles (OMVs) can affect inflammatory signaling, complement activity, vascular permeability, and neural-cell stress. Dominy and colleagues reported gingipain immunoreactivity in Alzheimer’s disease brain tissue and presented preclinical evidence that gingipain inhibition can reduce pathology-like changes in experimental models [7]. Importantly, this mechanistic lead did not translate into clinical benefit: the subsequent phase 2/3 GAIN trial of the gingipain inhibitor atuzaginstat (COR388) did not meet its co-primary cognitive and functional endpoints in the overall trial population, which underscores the gap between target engagement in model systems and disease modification in humans. Infected neuronal models also support persistent *P. gingivalis* virulence activity as a plausible cellular injury route [8].

Outer membrane vesicles are important because they allow bacterial virulence factors to act at a distance from intact bacteria. *P. gingivalis* vesicles can induce neuroinflammatory signaling in experimental models and may influence vascular permeability through endothelial mechanisms [9,10,11]. LPS-mediated pathways are also relevant, since *P. gingivalis* lipopolysaccharides can indirectly promote neuronal kinase activation and amyloidogenic or tau-relevant pathways in experimental systems [12]. These mechanisms are biologically credible, but the human dose, exposure duration, and brain availability remain unresolved.

*T. denticola* should not be ignored simply because it has fewer dedicated Alzheimer’s disease studies. Oral *Treponema* species have been detected in human brain tissue in association with Alzheimer’s disease [13], although such postmortem findings must be interpreted with caution given contamination and assay-specificity limitations [14,15]. Recent animal work further suggests that chronic oral inoculation with *P. gingivalis* and *T. denticola* can produce differential neurodegeneration-like changes, glial activation, and neuronal injury patterns [16].

*T. forsythia* has a strong periodontal virulence profile, but its direct neurodegenerative evidence remains weak. It expresses S-layer systems, BspA, GroEL, proteases, type IX secretion system (T9SS) substrates, and outer membrane vesicles that can shape host immune responses [17,18,19,20,21]. Toll-like receptor 2 (TLR2) signaling and Th2-skewed host responses are relevant to periodontal bone loss and inflammatory amplification [22,23]. However, these findings should not be extrapolated into a claim that *T. forsythia* is a confirmed brain pathogen. Its current status is best described as high periodontal plausibility with indirect neurobiological relevance. The organism-specific evidence is mapped in Table 2.

## 4. Biological Routes from the Periodontium to the Brain

Several biological routes could connect periodontal dysbiosis with brain aging (summarized in Table 3), but none should be interpreted as a complete causal chain in humans. The most credible are hematogenous spread of bacterial products and systemic immune signaling [24,25]; neural and oral–gut–brain routes remain more speculative, the latter lacking direct human evidence and presented here only as hypothesis-generating.

The blood–brain barrier is an important interpretive boundary: periodontal bacterial products can stress endothelial cells experimentally, but this does not mean intact periodontal pathogens routinely cross into human brain tissue. More cautiously, periodontal dysbiosis may contribute to barrier stress and immune amplification in susceptible hosts, especially alongside aging, diabetes, vascular disease, smoking, frailty, or apolipoprotein E (APOE)-related vulnerability. The APOE ε4, the strongest common genetic risk factor for late-onset Alzheimer’s disease, may plausibly interact with peripheral inflammatory burden, but this gene-by-inflammation interaction remains insufficiently tested in periodontal cohorts [26].

At the molecular level, the most plausible pathway is not direct microbial invasion but repeated peripheral inflammatory signaling, in which periodontal lipopolysaccharide, outer membrane vesicles, and proteases drive systemic release of mediators such as interleukin-1 beta (IL-1β), interleukin-6 (IL-6), tumor necrosis factor alpha (TNF-α), and C-reactive protein (CRP). This cascade should be interpreted as a testable mechanistic model, not as proof that oral bacterial products reach the human brain at clinically sufficient concentrations.

**Figure 2:**
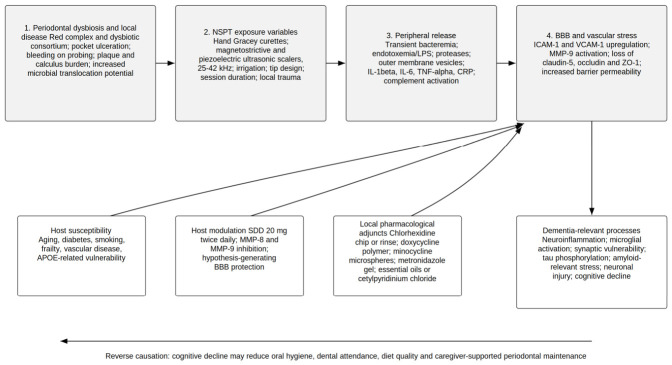
Bidirectional model of periodontal dysbiosis, host inflammatory susceptibility, clinical periodontal exposure variables, blood–brain barrier stress, and dementia-relevant biological processes. The schematic separates instrumentation modality, transient bacteremia and endotoxemia, host modulation, local pharmacological adjuncts, and blood–brain barrier mediators including intercellular adhesion molecule-1 (ICAM-1), vascular cell adhesion molecule-1 (VCAM-1), matrix metalloproteinase-9 (MMP-9), claudin-5, occludin, and zonula occludens-1. This is a conceptual model and not an empirically validated causal pathway diagram.

Directionality remains a core problem. Cognitive decline can reduce oral hygiene, reduce dental attendance, increase dependence on caregivers, change diet, and increase periodontal disease severity. Thus, the oral–brain association is likely bidirectional in clinical datasets. Any clinical interpretation that does not consider reverse causation will overestimate causal evidence from observational studies. This bidirectional model is depicted in Figure 2.

## 5. Human Association Evidence

Human evidence supports an association between periodontal disease, periodontal bacterial markers, and dementia-related outcomes but does not establish causality. In serological studies, elevated antibodies to periodontal bacteria preceded cognitive impairment by years [27], and serum IgG to periodontal microbiota was associated with the incidence of Alzheimer’s disease [28]. Such findings strengthen temporal plausibility but are directionally ambiguous, since raised titers may reflect either greater pathogen burden or a vigorous host immune response.

Large observational studies generally report that periodontitis, tooth loss, bacterial markers, or oral hygiene indicators are associated with dementia risk. A nationwide cohort described periodontitis as a modifiable dementia risk factor [29]. Clinical and bacterial markers of periodontitis were associated with incident all-cause and Alzheimer’s disease dementia in national survey data [30]. Infection burden and periodontal pathogens may also interact in association with incident all-cause and Alzheimer’s disease dementia [31].

Recent longitudinal evidence is valuable but still vulnerable to residual confounding. A 15-year cohort of older men in Northern Ireland reported associations between periodontitis, cognitive decline, and dementia [32]. Oral microbiome profiling studies in Alzheimer’s disease suggest dysbiotic shifts but remain mostly cross-sectional or associative [33,34]. Evidence from the All of Us cohort further shows why dental-care access must be handled carefully: unmet dental care needs due to cost were associated with incident dementia in less adjusted models, but the association was attenuated after additional adjustment for health status and periodontal disease, reinforcing the importance of socioeconomic and access-related confounding [35]. These findings support biological plausibility and health-equity relevance, but they do not resolve causality.

## 6. Periodontal Instrumentation and Host-Modulation: The Missing Clinical Variables

The clinically decisive question is not simply whether periodontal therapy was provided, but which therapy was provided, how it was delivered, whether it achieved periodontal resolution, and which local or systemic biological signals changed after treatment. Non-surgical periodontal therapy (NSPT) may include hand instrumentation, powered ultrasonic instrumentation, irrigation, local pharmacological adjuncts, antiseptic exposure, host modulation, and maintenance care. Treating these heterogeneous procedures as one exposure risks conflating mechanical biofilm disruption, transient bacteremia, endotoxemia, host-response modulation, local drug delivery, and access-to-care behavior. For dementia-relevant trials, periodontal therapy should therefore be decomposed into instrument type, treatment intensity, local pharmacological adjuncts, maintenance frequency, clinical periodontal response, and inflammatory or barrier-relevant biomarkers.

To reduce interpretive slippage, the exposures grouped in this section should be read as five distinct evidence categories, each supporting a different inferential statement. First, clinically defined periodontal treatment, such as protocol-described non-surgical therapy with recorded periodontal outcomes, is well established for oral-health endpoints, but its effect on dementia-relevant outcomes remains unproven. Second, self-reported or administrative dental-care exposure, such as treatment claims or utilization records, can support population-level associations only, because it captures care access and health-seeking behavior rather than biologically characterized treatment. Third, oral-hygiene behavior, such as tooth-brushing frequency, is best interpreted as a proxy for general health behavior and socioeconomic status rather than as a periodontal intervention. Fourth, adjunctive antiseptic intervention, most notably chlorhexidine, is a pharmacological adjunct and is not equivalent to non-surgical periodontal therapy or periodontal maintenance; evidence on antiseptics therefore speaks to local antimicrobial effects, not to comprehensive periodontal treatment. Fifth, biomarker-adjacent and imaging outcomes, such as brain-atrophy estimates or inflammatory markers, provide mechanistic and biomarker-level signals that are not interchangeable with a clinical dementia diagnosis. Read together, these categories indicate that periodontal therapy is established for oral health, whereas the dementia-related associations most plausibly reflect a mixture of biological effects, care access, adherence behavior, and socioeconomic confounding rather than a single demonstrated causal pathway.

### 6.1. Instrumentation Modality, Bacteremia, and Endotoxemia

NSPT includes hand instruments such as Gracey curettes and powered ultrasonic instruments. Magnetostrictive and piezoelectric ultrasonic scalers commonly operate in the ultrasonic range used clinically for dental debridement, approximately 25 to 42 kHz, but their biological effect is not determined by frequency alone. Tip design, lateral pressure, adaptation, irrigation, number of treated sites, pocket depth, bleeding on probing, calculus burden, session duration, and operator technique may all influence local trauma, lavage, aerosol generation, residual endotoxin, and the short post-procedural release of bacterial products. Clinical outcomes of ultrasonic and hand instrumentation are often broadly comparable, but the modalities are not mechanistically interchangeable [36]. Classic root-surface work showed that endotoxin values after ultrasonic scaling and meticulous hand root planing may differ under specific experimental conditions [37]. Clinical studies also show that scaling and root planing can cause transient bacteremia, usually peaking immediately after treatment and declining thereafter [38]. These observations do not prove that one instrumentation modality is neurologically safer than another, but they make instrumentation type an essential extractable variable in periodontal–brain research.

### 6.2. Host Modulation and Sub-Antimicrobial-Dose Doxycycline

Beyond mechanical and antiseptic approaches, host-modulation therapy is mechanistically relevant to the periodontal–brain question. Sub-antimicrobial-dose doxycycline (SDD; 20 mg twice daily, marketed as Periostat) is used as an adjunct to scaling and root planing and acts not as a conventional antibiotic but as a host-modulating agent that inhibits matrix metalloproteinases (MMPs), especially MMP-8 and MMP-9, thereby reducing periodontal tissue catabolism without relying on antibacterial dosing [39,40]. This is directly relevant to the biological model of this review because MMP-9 is also a recognized mediator of blood–brain barrier disruption. By degrading tight-junction and basement-membrane proteins such as claudin-5, occludin, and zonula occludens-1, MMP-9 can increase barrier permeability and contribute to neuroinflammatory amplification, and MMPs including MMP-9 are implicated in Alzheimer’s disease-related barrier dysfunction [41,42]. A periodontal therapy that lowers local or circulating MMP-9 activity is therefore a plausible translational bridge between periodontal inflammation and barrier protection. This remains a mechanistic hypothesis: no clinical trial has tested SDD, or any MMP-targeted periodontal adjunct, against cognitive, neuroimaging, amyloid, tau, neurofilament light chain, or blood–brain barrier endpoints.

### 6.3. Local Pharmacological Adjuncts

Local pharmacological adjuncts also require explicit extraction. Locally delivered periodontal agents include chlorhexidine chips, doxycycline polymer systems, minocycline microspheres or ointments, metronidazole gels, tetracycline fibers, and other controlled-release systems placed into periodontal pockets as adjuncts to mechanical debridement [43,44]. These agents can change local microbial load, pocket ecology, drug exposure, and inflammatory resolution, yet they are rarely distinguished in dementia-oriented observational studies. In a periodontal–brain trial, the use, dose, carrier, treated sites, residence time, number of applications, and adverse effects of local adjuncts should be recorded separately from mechanical instrumentation.

### 6.4. Chlorhexidine in Geriatric and Cognitively Impaired Populations

Cao et al. [33] add a clinically informative intervention-adjacent signal. In 100 patients with mild Alzheimer’s disease, 0.2% chlorhexidine gluconate was evaluated in relation to oral microbiota dysbiosis, with subgingival plaque analyzed by 16S rRNA sequencing and patients stratified by an oral-health score cut-off of 8. Chlorhexidine should be interpreted as a short-term adjunctive antiseptic and not as a substitute for NSPT or periodontal maintenance. It is a non-selective antiseptic and has limited subgingival penetration in deep pockets, and prolonged use is associated with tooth staining, taste disturbance or dysgeusia, mucosal irritation or desquamation, increased calculus formation and xerostomia [45,46]. In frail, geriatric or cognitively impaired patients, these effects may be clinically meaningful: dysgeusia can reduce food palatability and nutritional intake, mucosal desquamation and dry mouth can impair comfort and oral clearance, and impaired clearance may increase concern about aspiration. Because nutrition, frailty and aspiration risk can themselves influence cognitive and clinical outcomes, long-term chlorhexidine exposure could confound dementia trials. We therefore consider prolonged chlorhexidine use beyond a short defined indication inappropriate as a routine long-term intervention in dementia-focused studies. Future protocols should prioritize standardized mechanical debridement and maintenance and, where chemical adjuncts are justified, should pre-specify duration, monitoring and alternatives such as essential-oil or cetylpyridinium chloride formulations. Prolonged non-selective suppression may also promote oral dysbiosis, including relative overgrowth of Gram-negative rods, which is particularly important in frail older adults because microbial imbalance, impaired taste, reduced food intake and aspiration risk may confound cognitive outcomes.

### 6.5. Human Periodontal-Care Evidence and Translational Interpretation

Schwahn et al. [5] used a trial-emulation design comparing 177 periodontally treated patients with 409 untreated participants. After a median observation period of 7.3 years, periodontal treatment was associated with a favorable effect on Alzheimer’s disease-related brain atrophy, expressed as a standardized regression coefficient of −0.41 (95% confidence interval (CI) −0.70 to −0.12), where a negative value indicates less Alzheimer’s disease (AD)-pattern atrophy in treated participants. This finding is important because it links periodontal treatment exposure with a preclinical brain-aging marker, but the exposure still lacks sufficient granularity on instrumentation type, adjunctive therapy, periodontal response and inflammatory biomarker change. The key clinical studies of periodontal care and dementia-related outcomes, with bias-sensitive interpretation, are summarized in Table 4.

Qi et al. [6] analyzed 866 adults aged at least 50 years with periodontal symptoms in the Health and Retirement Study (HRS). During a median follow-up of 9 years, 105 participants developed dementia. Gum treatment was associated with lower dementia incidence, with event rates of 7.4 versus 12.9 per 1000 person-years, a slower annual decline in Telephone Interview for Cognitive Status (TICS) score by 0.025 points per year, 95% CI from 0.005 to 0.044, and a dementia hazard ratio (HR) of 0.62, 95% CI from 0.41 to 0.93. This is a clinically relevant prospective signal, but the exposure is dental-care based and does not specify whether treatment involved hand instrumentation, ultrasonic instrumentation, local adjuncts, SDD, maintenance adherence, probing pocket depth (PPD) reduction, bleeding on probing (BOP) reduction, clinical attachment level (CAL) gain, or biomarker normalization.

Administrative and population cohorts point in the same direction but remain especially sensitive to healthy-user bias. Periodontal treatment utilization was associated with lower dementia incidence in an older Japanese cohort [47]. In a Korean cohort of 2,555,618 individuals, professional dental cleaning and frequent tooth brushing were associated with lower dementia risk [48]. A more recent Korean analysis focused on moderate to severe periodontitis reported lower dementia risk among patients receiving regular dental scaling [49]. These studies are valuable because they are large and longitudinal, but treatment uptake and oral hygiene behavior may also proxy prevention-oriented care, socioeconomic advantage, caregiver support and better general health management.

**Figure 3:**
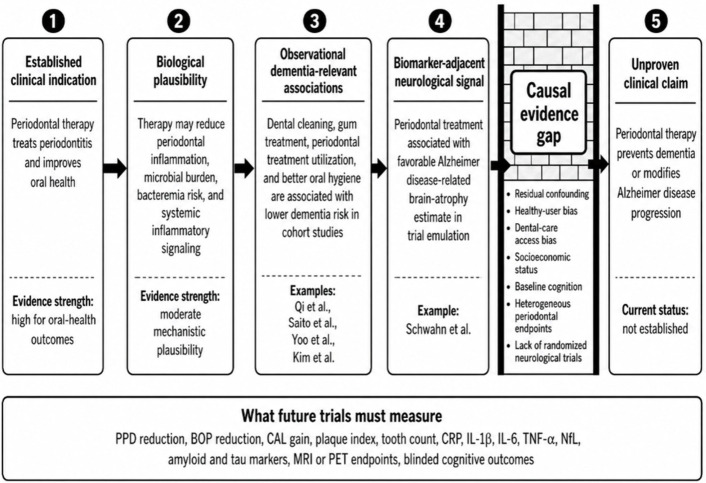
Author-derived conceptual model of the translational distance between established and unproven indications for periodontal therapy in relation to dementia. This is an interpretive framework reflecting the authors’ reading of the literature; it is not an evidence-certainty grade produced by a formal tool such as the Grading of Recommendations, Assessment, Development and Evaluations (GRADE) approach. Studies shown in the figure: Schwahn et al., 2022 [5]; Qi et al., 2024 [6]; Saito et al., 2022 [47]; Yoo et al., 2023 [48]; Kim et al., 2026 [49].

The clinically appropriate message is therefore deliberately narrow. Periodontal therapy is an established oral-health intervention and may reduce systemic inflammatory burden. Observational and quasi-experimental studies associate periodontal care with lower dementia risk or more favorable preclinical markers. However, periodontal therapy should not be presented as a proven dementia-preventive or disease-modifying intervention until randomized or rigorously emulated studies demonstrate effects on validated neurological endpoints while recording the missing clinical variables listed in Table 5. This translational distance between established and unproven indications is summarized in Figure 3.

## 7. Disease-Specific Interpretation and Scope Limitations

The evidence is strongest for Alzheimer’s disease and all-cause dementia, but outcome definitions vary across studies, from administrative diagnosis to cognitive testing and microbial or serological markers. These endpoints are not interchangeable and support different inferential claims. The primary outcome type reported by each key human study is summarized in Table 6, which makes explicit why these results were not pooled. Accordingly, the strongest disease-specific inferences—such as the brain-atrophy signal of Schwahn et al. [5] and the serological associations with incident Alzheimer’s disease of Noble et al. [28]—rest on Alzheimer’s disease-specific or biomarker-adjacent endpoints, whereas several of the larger associations (for example, the administrative-cohort studies of Lee et al. [29], Saito et al. [47], Yoo et al. [48], and Kim et al. [49]) are based on broader all-cause dementia or cognitive-decline outcomes. Conclusions framed at the level of Alzheimer’s disease should therefore be distinguished from those supported only by all-cause dementia or cognitive testing, and the present review treats the latter as more distal evidence.

The failure of the gingipain inhibitor atuzaginstat to meet its clinical endpoints, in contrast to clinically validated amyloid-targeting therapies [50,51], tempers expectations that targeting a single periodontal pathogen will modify cognition. A periodontal contribution to brain aging, if real, is therefore more plausibly an upstream or modifying inflammatory influence within a multifactorial disease than a standalone causal pathway.

Although Parkinson’s disease was included in the search strategy as contextual support for oral-systemic-neural plausibility, the scope and title were narrowed to Alzheimer’s disease and dementia, and Parkinson’s disease is not synthesized here.

The uneven organism-specific evidence also limits broad claims. *P. gingivalis* has mechanistic, postmortem, animal, cellular, serological, and clinical association support. *T. denticola* has biologically meaningful but less extensive support. *T. forsythia* has strong periodontal immunobiology but sparse direct neurodegenerative validation. A rigorous review must preserve this gradient rather than transferring the evidential strength of *P. gingivalis* to the entire red complex.

## 8. Research and Clinical Implications

Future studies should combine standardized periodontal phenotyping with pathogen-resolved microbiology and explicitly report numeric changes in periodontal, inflammatory and neurological markers. Clinical attachment loss, probing pocket depth, bleeding on probing, plaque index, tooth number, periodontal treatment history, maintenance adherence, instrumentation type, ultrasonic system, session duration, irrigation, operator protocol, SDD use, local antimicrobial carriers, antiseptic exposure, and chlorhexidine duration should be recorded at baseline and follow-up. For translational interpretation, periodontal changes should be correlated with CRP, IL-1beta, IL-6, TNF-alpha, MMP-8, MMP-9, neurofilament light chain (NfL), amyloid, tau, magnetic resonance imaging (MRI), positron emission tomography (PET), blood–brain barrier biomarkers and blinded cognitive outcomes. Without this level of reporting, clinicians cannot infer whether a dementia-relevant association reflects periodontal disease control, general care-seeking behavior, instrumentation-specific biological exposure, local pharmacological modulation or residual socioeconomic confounding. The extractable and currently missing clinical periodontal variables are detailed in Table 5.

Randomized periodontal trials with neurological endpoints are needed. A minimum design should include standardized non-surgical periodontal therapy, documented maintenance care, blinded cognitive assessment, inflammatory biomarkers, amyloid and tau markers, neurofilament light chain, barrier biomarkers, structural or molecular neuroimaging, adherence monitoring, and follow-up long enough to detect meaningful cognitive trajectories. Enrichment for mild cognitive impairment, amyloid positivity, high red complex burden, diabetes, vascular risk, or high systemic inflammation may increase biological sensitivity. The minimum requirements for such next-generation periodontal brain trials are listed in Table 7.

Clinical communication should remain precise. Dentists and physicians can state that periodontal therapy treats periodontitis and may reduce systemic inflammatory burden. They can also state that observational and quasi-experimental evidence links periodontal care with lower dementia risk. They should not present periodontal therapy as a validated dementia-modifying intervention. Financial barriers to dental care may contribute to dementia-related disparities, but this relation is partly confounded by socioeconomic status and access to preventive health care [35].

## 9. Limitations

This review is a narrative synthesis, not a registered systematic review and not a meta-analysis. Sources were selected purposively for thematic relevance rather than through formal eligibility screening, so the review does not claim exhaustive or reproducible source coverage and is subject to selection bias. The PubMed-centered search improves biomedical specificity but may miss dental, epidemiological, psychological, geriatric, and gray-literature sources indexed elsewhere. No protocol was prospectively registered, no duplicate independent screening was conducted, and no duplicate extraction was performed; the review therefore does not carry the evidential status of a systematic review.

A future systematic review on this question would begin with a registered protocol specifying the review question, databases, search dates, inclusion criteria, two-reviewer screening and full-text assessment, adjudication rules, a formal risk-of-bias tool, extractable periodontal variables, inflammatory biomarkers, neurological endpoints, and criteria for quantitative pooling. Only after that process would it be appropriate to pool treatment effects or to present clinical periodontal thresholds as evidence-based recommendations for dementia prevention.

The evidence base is heterogeneous across study design, exposure definition, pathogen assay, periodontal phenotype, dementia ascertainment, and follow-up duration. Most periodontal therapy evidence remains observational or quasi-experimental. Even carefully adjusted models cannot eliminate residual confounding by education, smoking, diabetes, frailty, income, diet, dental insurance, health literacy, medication use, and general health care engagement.

Several 2025 and 2026 studies are recent and require independent replication. The conclusions therefore emphasize biological plausibility and research priorities rather than clinical certainty. The review should be read as a narrative synthesis of a rapidly developing translational field, not as evidence that periodontal therapy is already established as dementia prevention.

## 10. Conclusions

The oral-microbiome–brain axis is biologically plausible and clinically relevant, but its evidential strength differs across pathogens and study designs. *P. gingivalis* remains the best-supported periodontal organism in relation to Alzheimer’s disease and dementia-relevant pathways. *T. denticola* is a credible candidate because of spirochetal biology, postmortem signals, neural-route hypotheses, and animal data. *T. forsythia* should be treated as a plausible inflammatory contributor rather than a confirmed neurodegenerative pathogen.

Standard periodontal therapy is justified for oral health and may plausibly reduce systemic inflammatory burden relevant to brain aging. Current prospective and quasi-experimental evidence suggests lower dementia risk or more favorable preclinical Alzheimer’s disease markers among treated individuals, but it does not prove dementia risk modification. The decisive next step is a randomized, biomarker-rich periodontal intervention trial with cognitive and neurodegenerative endpoints.

## Figures and Tables

**Table 1:** Scope of the review and key interpretive distinctions.

Element	Definition Used in This Review	Interpretive Consequence
Population	Adults and older adults, with emphasis on cohorts relevant to cognitive aging	Findings should not be generalized to children or acute neurological disorders
Exposure	Periodontitis, periodontal dysbiosis, red complex organisms, oral microbial markers, or periodontal care	Microbial exposure and treatment exposure are analyzed separately
Comparator	Lower periodontal burden, no reported periodontal treatment, or better oral hygiene status	Comparators are heterogeneous and often observational
Outcome	Alzheimer’s disease, all-cause dementia, cognitive decline, or dementia-relevant biological markers	Administrative dementia, biomarker-confirmed Alzheimer’s disease, and cognitive testing are not interchangeable
Scope boundary	Parkinson’s disease and other neurodegenerative disorders retained only as contextual evidence	Title and conclusions are restricted to Alzheimer’s disease and dementia

**Table 2:** Evidence map for red complex organisms in dementia-relevant mechanisms.

Organism	Periodontal Virulence	Brain-Relevant Evidence	Main Limitation	Judgment
*P. gingivalis*	Gingipains, LPS, OMVs, complement modulation	Postmortem markers, infected neurons, animal models, serology, cohorts	Human causal dose and timing remain unresolved	Strongest candidate among the reviewed periodontal organisms; no established clinical causality for dementia
*T. denticola*	Motility, dentilisin, Msp (major surface protein), spirochetal persistence	Oral *Treponema* in brain, trigeminal-route hypothesis, chronic inoculation model	Few longitudinal human datasets with species-resolved sampling	Moderate plausibility, limited validation
*T. forsythia*	S-layer, BspA, GroEL, KLIKK proteases, T9SS, OMVs	Immune and periodontal evidence, sparse direct brain evidence	Neurodegenerative evidence mostly inferential	Hypothesis-generating only

**Table 3:** Biological routes linking periodontal dysbiosis to dementia-relevant brain processes.

Route	Carrier or Signal	Potential Relevance	Major Uncertainty
Hematogenous	Bacteremia, LPS, proteases, OMVs	Endothelial activation, barrier stress, systemic inflammation	Human exposure dose and persistence
Neural	Trigeminal and olfactory connections	Potential craniofacial access route for microbial products or spirochetes	Direct trafficking evidence in humans
Immune	IL-1β, IL-6, TNF-α, CRP, complement	Microglial priming and neuroinflammatory amplification	Separation from aging and comorbidity
Oral-gut–brain	Swallowed bacteria, metabolites, gut dysbiosis	Systemic immune and metabolic signaling	Directionality and biomarker stability
Reverse direction	Cognitive decline, poor hygiene, reduced dental attendance	Dementia may worsen oral health	Confounding and reverse causation

**Table 4:** Key clinical studies of periodontal care and dementia-related outcomes with bias-sensitive interpretation.

Study	Design and Population	Exposure	Effect or Finding	Dominant Bias Domain	Bias-Sensitive Interpretation
[5]	Trial emulation, 177 treated and 409 untreated participants	Periodontal treatment	Favorable AD-related brain atrophy estimate, −0.41, 95% CI −0.70 to −0.12	Treatment selection, residual confounding	Important biomarker signal, but not randomized
[6]	HRS cohort, 866 adults aged at least 50 years with periodontal symptoms	Gum treatment	Dementia HR 0.62, 95% CI 0.41 to 0.93	Healthy-user bias, unmeasured dental-care access	Prospective association requiring trial confirmation
[47]	Japanese cohort, 31,775 older adults	Periodontal treatment utilization	More treatment days associated with lower dementia incidence	Administrative exposure, care-access confounding	Large population signal, not causal proof
[48]	Korean cohort, 2,555,618 individuals	Cleaning and tooth brushing	Lower dementia risk among individuals receiving oral hygiene care	Oral hygiene as proxy for general health behavior	Highly powered observational support
[49]	National Health Insurance Service (NHIS) cohort, moderate to severe periodontitis	Regular dental scaling	Lower dementia risk in weighted landmark analyses	Residual confounding despite robust modeling	Promising, but still nonrandomized

**Table 5:** Extractable clinical periodontal variables required in intervention-relevant evidence. The instrumentation and local pharmacological-adjunct columns separate mechanical debridement, host modulation, local drug delivery, and antiseptic exposure, thereby avoiding treatment of periodontal therapy as a single non-specific exposure.

Study or Future Design	Intervention or Exposure	Instrumentation Type	Local Pharmacological Adjuncts	Extractable or Missing Periodontal and Biomarker Parameters
Schwahn et al. [5]	Periodontal treatment in a trial-emulation framework; AD-related brain atrophy estimate −0.41, 95% CI −0.70 to −0.12	Not sufficiently specified; future extraction should distinguish hand Gracey curettes, ultrasonic instrumentation, session number, treated sites and maintenance	Not sufficiently specified; future extraction should record SDD, local antibiotics, chlorhexidine, essential oils, cetylpyridinium chloride and other adjuncts	Baseline and post-treatment PPD, BOP, CAL, plaque index, tooth count, maintenance adherence, CRP, IL-1beta, IL-6, TNF-alpha, MMP-8, MMP-9 and neurofilament light chain (NfL)
Qi et al. [6]	Gum treatment among adults with periodontal symptoms; dementia HR 0.62, 95% CI 0.41 to 0.93	Not specified; exposure should be separated into scaling, root planing, ultrasonic debridement, hand instrumentation and maintenance cleaning	Not specified; record SDD, local delivery systems, antiseptics and antibiotic exposure	Clinical periodontal response, treatment frequency, adherence, inflammatory biomarker response and dementia-relevant biomarker follow-up
Cao et al. [33]	0.2% chlorhexidine gluconate in mild Alzheimer’s disease with oral microbiota endpoint	Mechanical debridement not equivalent to chlorhexidine exposure and not sufficiently specified	Chlorhexidine gluconate; duration, adherence and adverse effects should be recorded explicitly	BOP, PPD, CAL, gingival index, plaque index, dysgeusia, mucosal desquamation, xerostomia, nutritional intake, aspiration events, CRP, IL-1beta, IL-6 and TNF-alpha
Future randomized periodontal–brain trial	Standardized NSPT plus maintenance versus control care	Predefine hand versus magnetostrictive ultrasonic versus piezoelectric ultrasonic instrumentation; record frequency range, tip type, irrigation, force, session duration and treated sites	Predefine SDD, local antimicrobials, chlorhexidine chip or rinse, essential oils, cetylpyridinium chloride, metronidazole gel, minocycline microspheres and other controlled-release carriers	Mean PPD reduction, percentage BOP reduction, CAL gain, plaque reduction, systemic cytokine change, MMP-8, MMP-9, NfL, amyloid, tau, magnetic resonance imaging (MRI) or positron emission tomography (PET), BBB markers and cognitive endpoints

**Table 6:** Primary outcome type reported by each key human study. The heterogeneity of outcomes—spanning administrative dementia diagnosis, Alzheimer’s disease diagnosis, cognitive test scores, serological markers, oral-microbiome profiles, and neuroimaging or brain-atrophy markers—is the main reason results were not pooled, because these endpoints are not statistically commensurable.

Study	Outcome as Reported	Outcome Category
Sparks Stein et al. [27]	Serum antibodies to periodontal bacteria preceding cognitive impairment	Serological marker/cognitive outcome
Noble et al. [28]	Serum IgG to periodontal microbiota; incident Alzheimer’s disease	Serological marker/Alzheimer’s disease diagnosis
Lee et al. [29]	Incident dementia in a nationwide cohort	Administrative dementia diagnosis
Beydoun et al. [30]	Incident all-cause and Alzheimer’s disease dementia (survey data)	All-cause and Alzheimer’s disease dementia diagnosis
Beydoun et al. [31]	Incident all-cause and Alzheimer’s disease dementia; infection burden	All-cause and Alzheimer’s disease dementia diagnosis
Farsi et al. [32]	Cognitive decline and incident dementia over 15 years	Cognitive test score/dementia diagnosis
Cao et al. [33]; Weber et al. [34]	Oral microbiota composition (16S rRNA profiling)	Oral microbiome profile
Velez et al. [35]	Incident dementia and unmet dental-care need	Administrative dementia diagnosis
Schwahn et al. [5]	Alzheimer’s disease-related brain-atrophy estimate	Neuroimaging/brain-atrophy marker (magnetic resonance imaging, MRI)
Qi et al. [6]	Dementia incidence (HR) and TICS cognitive score	Dementia diagnosis/cognitive test score
Saito et al. [47]	Dementia incidence and dental-care utilization	Administrative dementia diagnosis
Yoo et al. [48]	Dementia risk; cleaning and tooth-brushing exposure	Administrative dementia diagnosis
Kim et al. [49]	Dementia risk; regular dental scaling	Administrative dementia diagnosis

**Table 7:** Minimum requirements for next-generation periodontal brain trials.

Domain	Minimum Requirement	Reason
Periodontal phenotype	CAL, PPD, BOP, plaque index, tooth count, treatment history	Prevents vague exposure definitions
Microbiology	Species-resolved oral sampling, OMVs, LPS, proteases, antibody profiles	Separates *P. gingivalis*, *T. denticola*, and *T. forsythia*
Neurology	Blinded cognition, amyloid, tau, NfL, MRI or PET, barrier biomarkers	Links oral intervention to brain-relevant endpoints
Design	Randomized NSPT plus maintenance, adherence monitoring, long follow-up	Tests causality rather than care-seeking behavior
Confounding	APOE, diabetes, smoking, education, frailty, dental access, income	Reduces socioeconomic and healthy-user bias

## Data Availability

No new datasets were generated. All data analyzed during this review are derived from previously published sources cited within the article.

## Appendix A. PubMed Search Strings and Narrative Source-Selection Log

Search date: 4 May 2026. Database: PubMed. Appendix A documents the full PubMed search strings, the query-count table, and the narrative screening log within the manuscript. Because this is a narrative review rather than a registered systematic review, the query-count table reports the number of sources retained after query-level narrative screening and the corresponding retained citation mapping; these counts are not PRISMA eligibility counts and should not be interpreted as formal database-yield statistics.

Narrative screening log summary: Twelve PubMed query groups were reviewed for thematic relevance. Forty-six periodontal-scope sources were retained in the synthesis. Five contextual non-periodontal references were retained outside the periodontal PubMed search because they were required for dementia epidemiology, Alzheimer’s disease trial context, and blood–brain barrier biology. The query-level counts in Table A1 identify how the retained sources were mapped to the search concepts used in the manuscript.

**Table A1:** PubMed query groups, full search strings, query-level counts, and narrative screening log retained within the manuscript.

Group	Full PubMed Search String	Query Count After Narrative Screening and Retained Citation Numbers
Q1	(periodontitis [Title/Abstract] OR “periodontal disease” [Title/Abstract]) AND (“Alzheimer disease” [Title/Abstract] OR dementia [Title/Abstract] OR “cognitive decline” [Title/Abstract])	Query-level retained count: *n* = 14; Core association and cohort sources: [5,6,27,28,29,30,31,32,33,34,35,47,48,49]
Q2	(“oral microbiome” [Title/Abstract] OR “oral microbiota” [Title/Abstract] OR “oral dysbiosis” [Title/Abstract]) AND (“Alzheimer disease” [Title/Abstract] OR dementia [Title/Abstract])	Query-level retained count: *n* = 8; Oral microbiome and dysbiosis sources: [33,34] plus mechanistic context [7,8,9,10,11,12]
Q3	(“*Porphyromonas gingivalis*” [Title/Abstract] OR gingipain* [Title/Abstract]) AND (“Alzheimer disease” [Title/Abstract] OR dementia [Title/Abstract] OR neuroinflammation [Title/Abstract])	Query-level retained count: *n* = 11; *P. gingivalis* and gingipain sources: [7,8,9,10,11,12,27,28,29,30,31]
Q4	(“*Treponema denticola*” [Title/Abstract] OR “oral Treponema” [Title/Abstract]) AND (“Alzheimer disease” [Title/Abstract] OR dementia [Title/Abstract] OR brain [Title/Abstract])	Query-level retained count: *n* = 4; *T. denticola* and oral spirochete sources: [13,14,15,16]
Q5	(“*Tannerella forsythia*” [Title/Abstract] OR “Bacteroides forsythus” [Title/Abstract]) AND (periodontitis [Title/Abstract] OR inflammation [Title/Abstract] OR neuroinflammation [Title/Abstract] OR dementia [Title/Abstract])	Query-level retained count: *n* = 7; *T. forsythia* periodontal virulence and immune sources: [17,18,19,20,21,22,23]
Q6	(“red complex” [Title/Abstract] OR “periodontal pathogen*” [Title/Abstract]) AND (dementia [Title/Abstract] OR “Alzheimer disease” [Title/Abstract] OR neuroinflammation [Title/Abstract])	Query-level retained count: *n* = 10; Red complex and pathogen consortium sources: [4,17,18,19,20,21,22,23,30,31]
Q7	(“periodontal treatment” [Title/Abstract] OR “periodontal therapy” [Title/Abstract] OR “gum treatment” [Title/Abstract] OR “dental scaling” [Title/Abstract]) AND (dementia [Title/Abstract] OR “cognitive decline” [Title/Abstract] OR “Alzheimer disease” [Title/Abstract])	Query-level retained count: *n* = 5; Treatment and dental-care sources: [5,6,47,48,49]
Q8	(chlorhexidine [Title/Abstract] OR “chlorhexidine gluconate” [Title/Abstract] OR CHX [Title/Abstract]) AND (“Alzheimer disease” [Title/Abstract] OR dementia [Title/Abstract] OR geriatric [Title/Abstract] OR “oral microbiota” [Title/Abstract])	Query-level retained count: *n* = 3; Chlorhexidine and geriatric caution sources: [33,45,46]
Q9	(“sub-antimicrobial dose doxycycline” [Title/Abstract] OR “subantimicrobial dose doxycycline” [Title/Abstract] OR Periostat [Title/Abstract] OR doxycycline [Title/Abstract]) AND (periodontitis [Title/Abstract] OR “matrix metalloproteinase” [Title/Abstract] OR MMP-8 [Title/Abstract] OR MMP-9 [Title/Abstract])	Query-level retained count: *n* = 2; Host-modulation and SDD sources: [39,40]
Q10	(“MMP-9” [Title/Abstract] OR “matrix metalloproteinase-9” [Title/Abstract]) AND (“blood–brain barrier” [Title/Abstract] OR “Alzheimer disease” [Title/Abstract] OR neuroinflammation [Title/Abstract])	Query-level retained count: *n* = 2; BBB and MMP context sources: [41,42]
Q11	((scaling [Title/Abstract] OR “root planing” [Title/Abstract] OR “ultrasonic” [Title/Abstract] OR “hand instrumentation” [Title/Abstract] OR Gracey [Title/Abstract]) AND (bacteremia [Title/Abstract] OR endotoxin [Title/Abstract] OR endotoxemia [Title/Abstract] OR instrumentation [Title/Abstract]))	Query-level retained count: *n* = 3; Instrumentation, bacteremia and endotoxin sources: [36,37,38]
Q12	((“local drug delivery” [Title/Abstract] OR “local antimicrobial” [Title/Abstract] OR “chlorhexidine chip” [Title/Abstract] OR “minocycline microspheres” [Title/Abstract] OR “metronidazole gel” [Title/Abstract] OR “doxycycline polymer” [Title/Abstract]) AND periodontitis [Title/Abstract])	Query-level retained count: *n* = 2; Local pharmacological adjunct sources: [43,44]

Note: The asterisk (*) within search terms (e.g., “gingipain*”) denotes the PubMed truncation (wildcard) operator, which retrieves all word endings of the stem; it is part of the search syntax and is not a reference marker or footnote symbol.
